# NEIL3 and TOP2A as key drivers of esophageal cancer through WNT signaling

**DOI:** 10.17305/bb.2025.11365

**Published:** 2025-01-29

**Authors:** Hui Li, Panpan Wang, Huijuan Chen, Yanyan Shao, Hui Luo

**Affiliations:** 1 Tumor Medical Center, The First Affiliated Hospital, Jiangxi Medical College of Nanchang University, Nanchang, China; 2 Department of Oncology, The First Hospital of Nanchang, Nanchang, China

**Keywords:** Esophageal cancer, EC, nei like DNA glycosylase 3, NEIL3, DNA topoisomerase II alpha, TOP2A, WNT signaling pathway, apoptosis

## Abstract

Esophageal cancer (EC) is a highly aggressive malignancy with limited treatment options. Nei like DNA glycosylase 3 (NEIL3) and DNA topoisomerase II alpha (TOP2A) have been identified as potential therapeutic targets, though their roles in EC remain unclear. This study investigates the effects of NEIL3 overexpression and TOP2A knockdown, focusing on the WNT signaling pathway. ECA109 esophageal cancer cells were used to assess the impact of NEIL3 overexpression and TOP2A knockdown on proliferation, colony formation, migration, invasion, and apoptosis. The involvement of the WNT signaling pathway was also explored. NEIL3 overexpression significantly enhanced proliferation, colony formation, migration, and invasion while reducing apoptosis. In contrast, TOP2A knockdown suppressed these functions and promoted apoptosis, independent of NEIL3. NEIL3 overexpression could not reverse the effects of TOP2A knockdown. Both NEIL3 and TOP2A acted through the WNT signaling pathway. *In vivo*, NEIL3 knockdown reduced tumor size and weight via WNT pathway modulation. NEIL3 and TOP2A play key roles in EC progression through the WNT signaling pathway. Targeting these molecules may offer promising therapeutic strategies for EC.

## Introduction

Esophageal cancer (EC) is a malignant neoplasm arising from the esophageal epithelium and remains a significant challenge in oncology due to its aggressive nature and limited treatment options [1, 2]. This cancer type contributes substantially to the global health burden, with incidence rates varying across different geographical regions. The etiology of EC is multifactorial, involving genetic susceptibility, environmental exposures, and lifestyle factors. Major risk factors include prolonged exposure to carcinogens, such as tobacco smoke and alcohol, which induce DNA damage and promote tumorigenesis [3, 4]. Despite advancements in early diagnosis and treatment, the molecular mechanisms underlying EC remain a key area of research [5]. Understanding these mechanisms is essential for developing targeted and personalized therapies. Recent studies highlight the WNT signaling pathway as a critical player in EC pathogenesis [6]. This pathway regulates cell proliferation, differentiation, and migration and plays a vital role in modulating the tumor microenvironment [7]. Nei like DNA glycosylase 3 (NEIL3), a DNA glycosylase involved in repairing oxidative DNA damage, is crucial for maintaining genomic stability [8]. Its expression is closely linked to cellular resistance to oxidative stress, and its functional loss may contribute to tumorigenesis [9]. In colorectal cancer, NEIL3 expression is regulated by the WNT signaling pathway, influencing DNA repair capacity [10]. However, its role in EC progression via WNT signaling remains unclear. DNA topoisomerase II alpha (TOP2A) is an enzyme essential for DNA replication and transcription, resolving DNA supercoiling through strand cleavage and re-ligation [11]. Abnormal TOP2A expression is associated with various cancers, particularly in rapidly proliferating tumor cells, where its levels are often significantly elevated [12]. It is not only involved in cell cycle regulation but also plays a role in chemoresistance [13]. In EC, TOP2A activity may be regulated by the WNT signaling pathway, impacting tumor cell proliferation and survival [14]. Our previous STRING database analysis suggested a potential regulatory association between NEIL3 and TOP2A. This study aims to investigate their relationship and role in EC progression through the WNT signaling pathway. By elucidating the involvement of NEIL3 and TOP2A in WNT signaling, we hope to provide new insights into EC pathogenesis and identify potential therapeutic targets for early diagnosis and treatment.

## Materials and methods

### Cell culture

Normal esophageal epithelial cells (HEEpiC) and EC cell lines (TE-1, ECA-109, OE21) were cultured in Dulbecco’s Modified Eagle Medium (DMEM) supplemented with 10% fetal bovine serum (FBS) and 1% penicillin–streptomycin. For NEIL3 overexpression, ECA109 cells were seeded in culture plates and grown to 70%–80% confluence. Cells were then transfected with a NEIL3-expressing plasmid (oeNEIL3) from Tsingke Biotechnology Co., Ltd., China, or an empty vector (pcDNA3.1) using Lipofectamine™ 3000, following the manufacturer’s protocol. To knock down DNA topoisomerase 2-alpha (TOP2A), ECA109 cells were transfected with TOP2A siRNA (siTOP2A) using Lipofectamine™ 3000, in accordance with the manufacturer’s instructions [15].

### Quantitative real-time polymerase chain reaction (qRT–PCR)

We obtained NEIL3-expressing plasmid (oeNRIL3) transfected cells and TOP2AsiRNA from Tsingke Biotechnology Co., Ltd. (Beijing, China). The sequences of siRNAs applied are shown as following: NC (negative control): Sense: 5-AUUGACCUAACUUUGGAGCGUAU-3, Antisense: 5-ACGCUCCAAAGUUAGGUCAAU-3; NEIL3 siRNA: Sense: 5-UGAUGAAGCCUUUCAUUCCGA-3, Antisense: 5-GGAAUGAAAGGCUUCAUCAUG-3; TOP2A siRNA: Sense: 5-UGCAUAUUUUCAUUUACAGGC-3, Antisense: 5-CUGUAAAUGAAAAUAUGCAAG-3. After transfection of EC cells, total RNA was extracted using TRIzol reagent (SuperfecTRI, China) and reverse transcribed into cDNA using Promega M-MLV (Beijing, China). The resulting cDNA was analyzed by qRT–PCR (Takara, Dalian, China) on an Agilent Real-Time PCR System (Agilent, CA, USA). The primers used in this study were as follows: TBRG4: 5-CAGCTCACCTGGTAAAGCGAT-3 (forward) and 5-GGGAGTAGATGCTCGTTCCTTC-3 (reverse); GAPDH: 5-TGACTTCAACAGCGACACCCA-3 (forward) and 5-CACCCTGTTGCTGTAGCCAAA-3 (reverse).

### CCK-8 assay

Following NEIL3 overexpression and/or TOP2A knockdown, cell proliferation was assessed using the CCK-8 assay. Briefly, transfected cells were seeded into 96-well plates at appropriate densities and cultured for predetermined time points. At each time point, CCK-8 solution was added to the wells and incubated at 37 ^∘^C for 1–4 h. Absorbance was then measured at 450 nm using a microplate reader.

### Clonogenic assay

Following NEIL3 overexpression and/or TOP2A knockdown, clonogenic ability was assessed using a clonogenic assay. Single-cell suspensions were prepared by trypsinization and counted using a hemocytometer. Cells were then seeded at appropriate dilutions in culture dishes and incubated for 10–14 days at 37 ^∘^C in a humidified atmosphere with 5% CO_2_ to allow colony formation. After incubation, colonies were fixed with methanol, stained with crystal violet, and counted under a light microscope, with only colonies containing at least 50 cells included in the analysis.

### Transwell chamber assay

Cell migration and invasion assays were performed using Transwell chambers. Transfected ECA109 cells (1 × 10^5^ in serum-free medium) were seeded into the upper chamber, which was either coated with Matrigel to simulate the extracellular matrix or left uncoated. The lower chamber contained medium supplemented with 10% FBS. After 24–48 h of incubation, non-migrated or non-invaded cells on the upper membrane surface were removed, while migrated or invaded cells on the lower surface were fixed, stained, and counted under a microscope [16].

### Annexin V staining

Apoptosis was assessed using Annexin V staining according to the manufacturer’s instructions (Annexin V-FITC Apoptosis Detection Kit, BD Biosciences, Catalog No. 556547). Transfected ECA109 cells were harvested, washed with phosphate-buffered saline (PBS), and resuspended in Annexin V binding buffer (BD Biosciences, Catalog No. 556454). The cells were then stained with Annexin V-FITC and propidium iodide (PI) for 15 min at room temperature in the dark. Immediately after staining, apoptotic populations were quantified using flow cytometry (BD LSRFortessa, BD Biosciences).

### Western blot analysis

Protein was extracted from ECA109 cells or mouse tissue using RIPA lysis buffer (Solarbio, Beijing, China) following previously described protocols [17]. Protein concentration was measured using a BCA assay kit (Beyotime, Shanghai, China) according to the manufacturer’s instructions. Equal amounts of protein were separated by SDS-PAGE and transferred onto PVDF membranes (Merck Millipore, USA). The membranes were then blocked with 5% non-fat milk or BSA and incubated overnight at 4 ^∘^C with primary antibodies targeting key proteins in the WNT signaling pathway, including β-catenin, cyclin D1, and TCF. After washing, the membranes were incubated with HRP-conjugated secondary antibodies (Abcam, 1:1000, Rabbit). Protein bands were visualized using an enhanced chemiluminescence (ECL) detection system, and protein expression levels were quantified using densitometric analysis software. Antibody information: Anti-β-Catenin (Abcam, ab32572, 1:10,000 dilution, Rabbit) Anti-Cyclin D1 (Abcam, ab134175, 1:100,000 dilution, Rabbit) Anti-HCF-1 (Abcam, ab137618, 1:500 dilution, Rabbit).

### Nude mouse xenograft model

Nude mice were housed under standard laboratory conditions with ad libitum access to food and water. ECA109 cells infected with either NC or NEIL3 lentivirus (General Biol, China) were harvested, resuspended in PBS, and prepared for injection. A total of 100 µL of ECA109 cells (1 × 10^6^) in PBS was subcutaneously injected into the flank region of each nude mouse. Tumor growth was monitored regularly, and tumor size was measured using calipers. After 20 days, the mice were euthanized, and the tumors were excised. Tumor size was measured again using calipers, and tumor volume was calculated using the formula: volume ═ (length × width^2^) × 0.5. Tumor tissues were then weighed using a precision balance.

### Ethical statement

The experimental protocols were approved by the Ethics Committee of The First Affiliated Hospital, Jiangxi Medical College of Nanchang University (Approve number: CDYFY-IACUC-202407QR204).

### Statistical analysis

GraphPad Prism v9.0 was used for data analysis and visualization. Data are presented as the mean ± standard deviation (SD). A one-way analysis of variance (ANOVA) was performed to evaluate differences among multiple groups, followed by the Least Significant Difference (LSD) test. A *P* value of <0.05 was considered statistically significant.

## Results

### TOP2A and NEIL3 in EC cell proliferation

**Figure 1.**
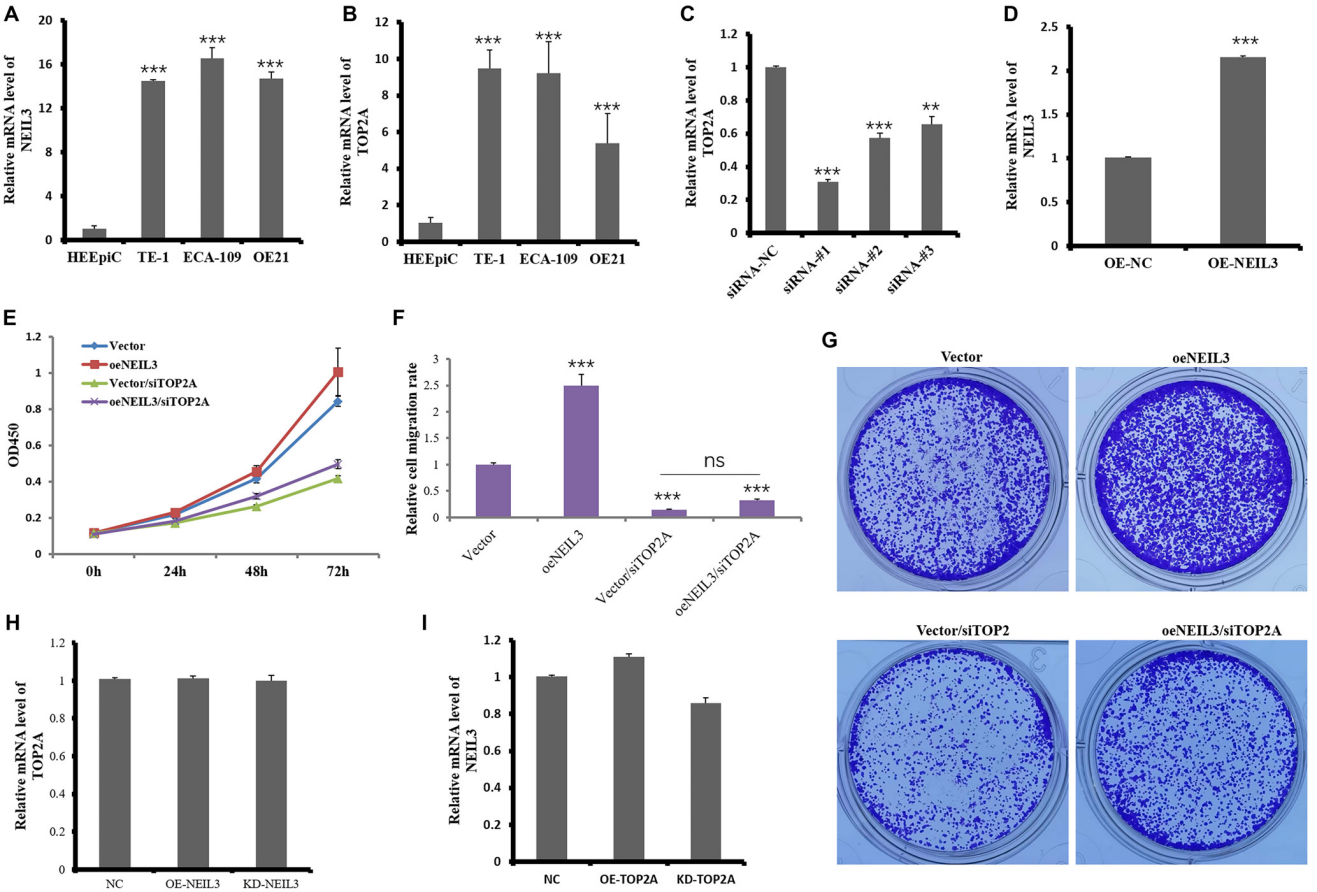
**TOP2A mediates NEIL3-induced cell proliferation in EC cells.** ECA109 cells were transfected with TOP2A siRNA for 6 h, followed by the transfection of NEIL3 or vector plasmid. At different time points, cells were detached and used for CCK-8 assay. qRT-PCR was used to detect the expression levels of (A) NEIL3 and (B) TOP2A in normal esophageal epithelial cell lines (HEEpiC) and esophageal cancer cell lines (TE-1, ECA-109, OE21). qRT-PCR detection of (C) TOP2A knockout and (D) NEIL3 overexpression effects. (E) Effects of NEIL3 overexpression and TOP2A knockdown on cell proliferation were assessed at various time points (0, 24, 48, and 72 h) using the CCK-8 assay. Cell clone results indicated effects of NEIL3 overexpression and TOP2A knockdown on clone formation (F) and their analysis (G). (H) qRT-PCR detection of TOP2A expression level after NEIL3 overexpression and knockout. (I) qRT-PCR detection of NEIL3 expression level after TOP2A overexpression and knockout. ***P < 0.001. significant difference, compared to vector. EC: Esophageal cancer; TOP2A: Topoisomerase II alpha; NEIL3: Nei like DNA glycosylase 3; qRT-PCR: Quantitative real-time polymerase chain reaction.

Understanding the regulatory mechanisms of cell proliferation in cancer is critical for developing effective therapeutic strategies. We first assessed NEIL3 and TOP2A expression levels in an EC cell line and a non-tumor human esophageal epithelial cell line using qPCR. The results confirmed that both NEIL3 and TOP2A were significantly upregulated in the EC cell line (Figure 1A and B).

Next, their effects on EC cell proliferation were studied by overexpressing NEIL3 or knocking down TOP2A in ECA109. The results are shown in Figure 1C and 1D. We successfully overexpressed NEIL3 and knocked down TOP2A, which can be used for subsequent cell function verification. CCK8 results showed that NEIL3 overexpression significantly increased cell proliferation, while siRNA-mediated TOP2A knockdown significantly reduced cell proliferation. However, NEIL3 overexpression did not reverse the inhibitory effect of TOP2A knockdown on cell proliferation (Figure 1E). Colony formation assays revealed that NEIL3 overexpression led to a significant increase in the number of cell colonies formed. In contrast, TOP2A downregulation resulted in a significant decrease in colony formation. Moreover, the inhibitory effect of TOP2A knockdown on colony formation could not be reversed by NEIL3 overexpression (Figure 1F and 1G).

**Figure 2.**
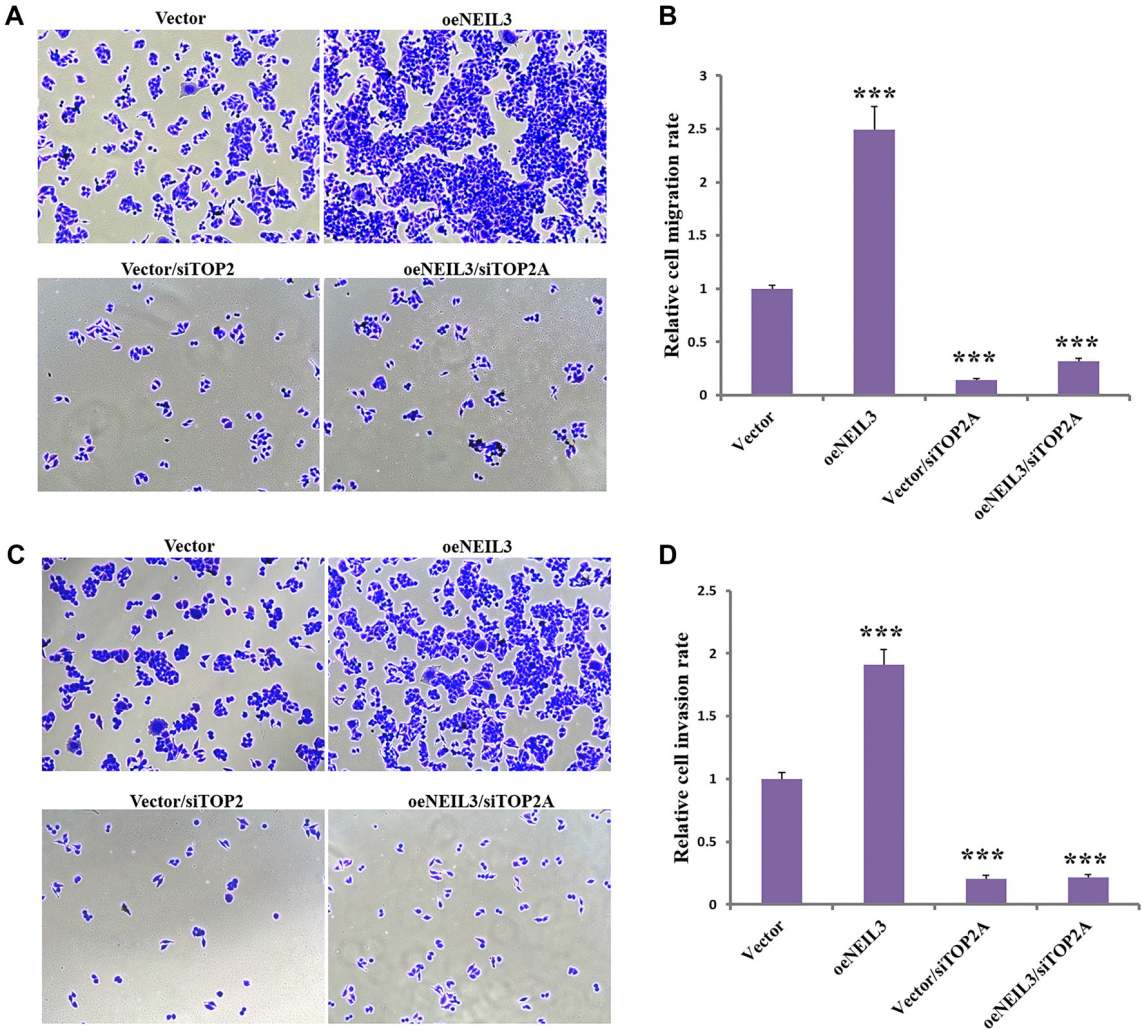
**TOP2A mediates NEIL3-induced cell migration and invasion in EC cells.** ECA109 cells were transfected with TOP2A siRNA for 6 h, followed by the transfection of NEIL3 or vector plasmid. Transwell chamber data indicated effects of NEIL3 overexpression and TOP2A knockdown on cell migration (A) and their analysis (B). Transwell chamber data indicated effects of NEIL3 overexpression and TOP2A knockdown on cell invasion (C) and their analysis (D). ***P < 0.001. significant difference, compared to vector. EC: Esophageal cancer; TOP2A: Topoisomerase II alpha; NEIL3: Nei like DNA glycosylase 3.

In our previous analysis using the STRING database, we identified a potential association between NEIL3 and TOP2A, prompting us to further explore their relationship. To investigate whether NEIL3 overexpression or knockdown affected TOP2A expression levels, we were surprised to find that TOP2A expression remained unchanged (Figure 1H). Similarly, TOP2A overexpression did not alter NEIL3 expression levels. However, TOP2A knockdown slightly reduced NEIL3 expression (*P* > 0.05) (Figure 1I). We believe that TOP2A knockdown may cause DNA replication stress and proliferation inhibition, which may affect NEIL3 expression by activating cellular response mechanisms (such as DNA damage response, cell cycle checkpoints, etc.). Although both NEIL3 and TOP2A play key roles in DNA metabolism, our results suggest that their roles in EC cell proliferation may be regulated by independent mechanisms rather than being interdependent.

### NEIL3 and TOP2A in regulating migration and invasion in EC cells

Understanding the impact of NEIL3 and TOP2A on cell migration and invasion is crucial for defining their roles in EC. Since migration and invasion are key hallmarks of cancer that drive tumor progression and metastasis, we investigated how NEIL3 and TOP2A influence these processes in EC cells. Consistent with our previous findings, NEIL3 overexpression significantly increased the number of migrating (Figure 2A and 2B) and invading cells (Figure 2C and 2D), whereas TOP2A knockdown markedly reduced both. However, NEIL3 overexpression did not counteract the inhibitory effect of TOP2A knockdown on migration and invasion (Figure 2A–2D). This suggests that the effects of TOP2A on cell behavior may be independent of NEIL3 or that NEIL3 is not a direct regulator of these processes. Further investigation into the molecular mechanisms underlying these interactions could provide valuable insights for developing targeted therapies to inhibit cancer invasion and metastasis.

### NEIL3 and TOP2A affect apoptosis of EC cells

**Figure 3.**
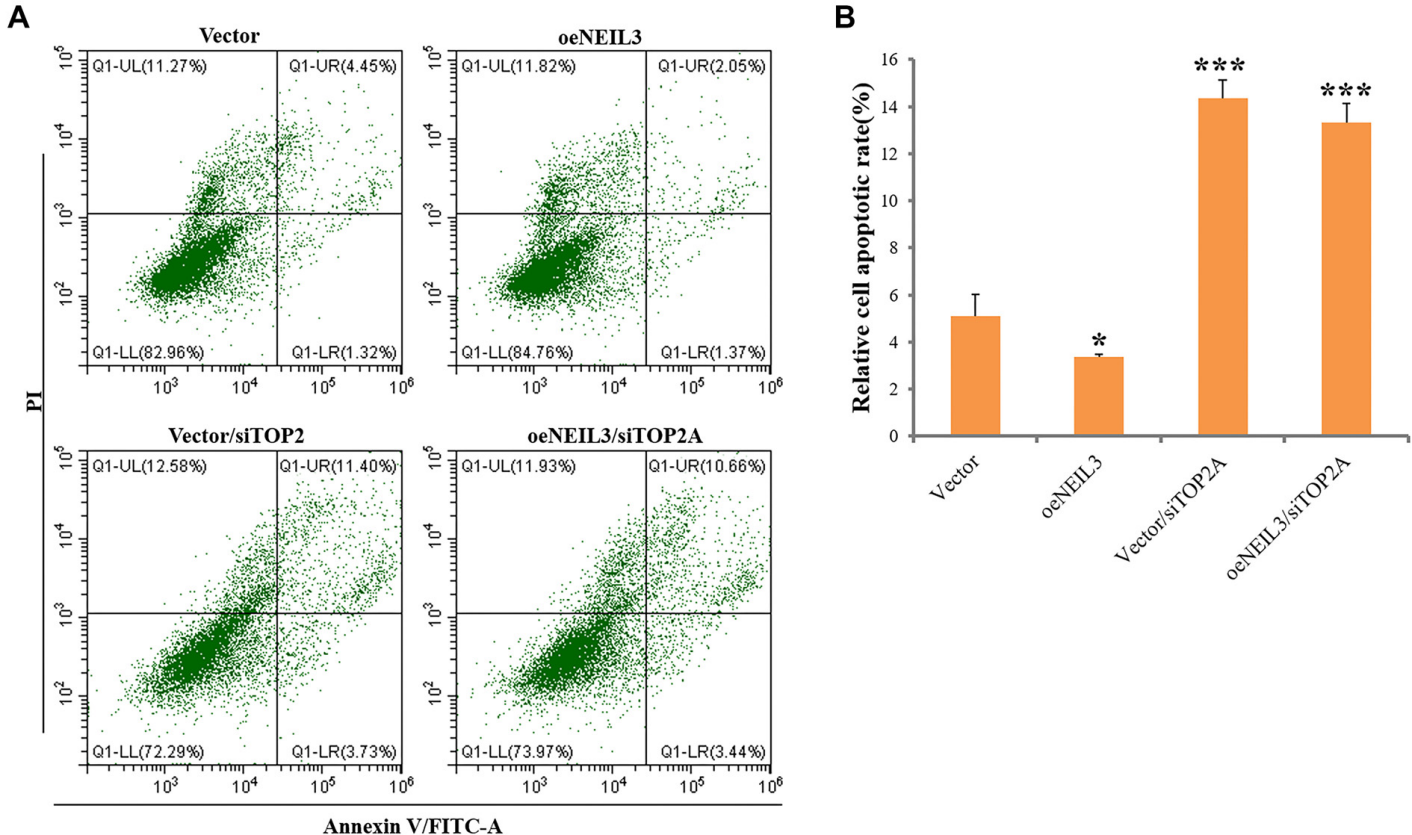
**TOP2A mediates NEIL3-suppressed cell apoptosis in EC cells.** ECA109 cells were transfected with TOP2A siRNA for 6 h, followed by the transfection of NEIL3 or vector plasmid Annexin V results indicated effects of NEIL3 overexpression and TOP2A knockdown on cell apoptosis (A) and their analysis (B). *P < 0.05, *** *P* <0.001. significant difference, compared to vector. EC: Esophageal cancer; TOP2A: Topoisomerase II alpha; NEIL3: Nei like DNA glycosylase 3.

Apoptosis, or programmed cell death, is essential for maintaining tissue homeostasis and suppressing tumorigenesis. In this study, we examined the roles of NEIL3 and TOP2A in regulating apoptosis in EC cells. We found that NEIL3 overexpression significantly reduced the number of apoptotic cells, effectively inhibiting apoptosis, whereas TOP2A knockdown markedly increased apoptotic cell death. However, NEIL3 overexpression did not affect TOP2A-induced apoptosis (Figure 3A and 3B). These findings highlight the distinct roles of NEIL3 and TOP2A in apoptosis regulation, suggesting that further investigation into their molecular mechanisms could reveal potential therapeutic targets for promoting apoptosis and limiting cancer cell survival in EC.

### NEIL3 and TOP2A in regulating WNT signaling

**Figure 4.**
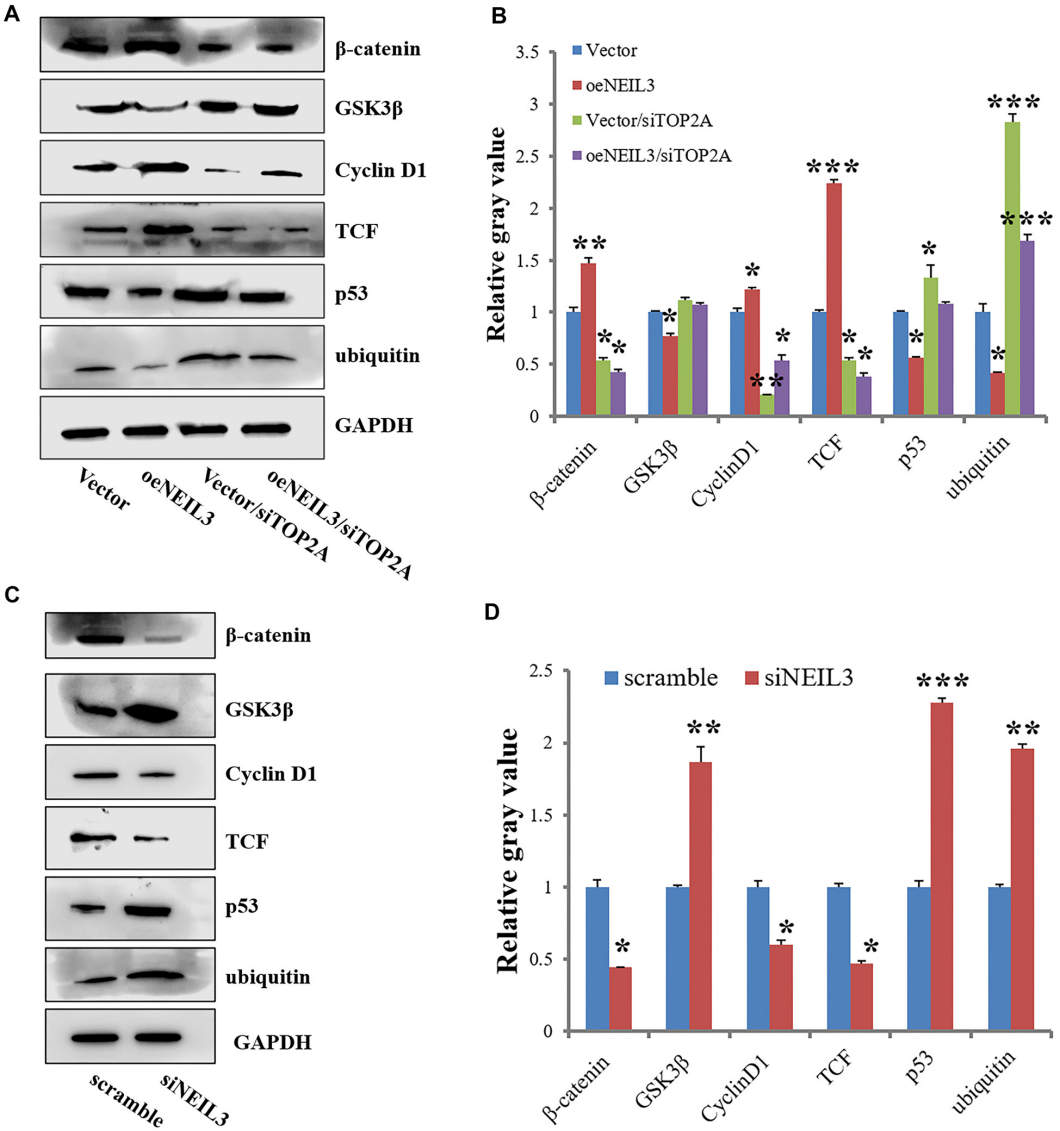
**NEIL3 and TOP2A in regulating WNT signaling.** ECA109 cells were transfected with TOP2A siRNA for 6 h, followed by the transfection of NEIL3 or vector plasmid Western Blot results indicated effects of NEIL3 overexpression and TOP2A knockdown on WNT signaling pathway (A) and their analysis (B). *P < 0.05. Significant difference, compared to vector. ECA109 cells were transfected with scramble siRNA or NEIL3 siRNA for 48 h, then Western blot results tested their effect on WNT signaling pathway (C) as well as their data analysis (D). *P < 0.05, **P < 0.01, ***P < 0.001. Significant difference, compared to scramble. TOP2A: Topoisomerase II alpha; NEIL3: Nei like DNA glycosylase 3.

Recent studies suggest that the WNT signaling pathway may play a role in DNA damage repair, particularly in certain tumors [18]. Additionally, research indicates that activation of the WNT signaling pathway can influence the expression of DNA repair genes, including NEIL3 [10]. This implies that the WNT pathway may modulate DNA repair processes by regulating genes such as NEIL3. Therefore, we investigated whether the WNT signaling pathway regulates NEIL3 and TOP2A in EC. Overexpression of NEIL3 significantly increased the expression of β-catenin, TCF, and cyclin D1, while TOP2A knockdown decreased the protein levels of these three factors. However, NEIL3 overexpression did not prevent the reduction in β-catenin, TCF, and cyclin D1 protein levels induced by TOP2A knockdown. Similarly, NEIL3 overexpression reduced the levels of glycogen synthase kinase 3β (GSK3β), tumor protein p53, and ubiquitin, whereas TOP2A knockdown significantly increased p53 and ubiquitin expression but had no effect on GSK3β levels (Figure 4A and 4B). Furthermore, although NEIL3 overexpression partially reversed the TOP2A knockdown-induced increase in ubiquitin levels, ubiquitin expression remained higher than control levels. Interestingly, NEIL3 overexpression also counteracted the increase in p53 expression caused by TOP2A knockdown (Figure 4A and 4B). Conversely, NEIL3 knockdown resulted in a significant increase in β-catenin, cyclin D1, and TCF protein levels—key components of the WNT signaling pathway. At the same time, NEIL3 knockdown led to a substantial decrease in GSK3β, p53, and ubiquitin protein expression (Figure 4C and 4D). These findings suggest that the WNT signaling pathway is involved in this regulatory process, highlighting a complex interplay between NEIL3, TOP2A, and the WNT pathway in controlling protein expression in EC cells.

### Knockdown NEIL3 reduced tumor growth through WNT signaling pathway *in vivo*

**Figure 5.**
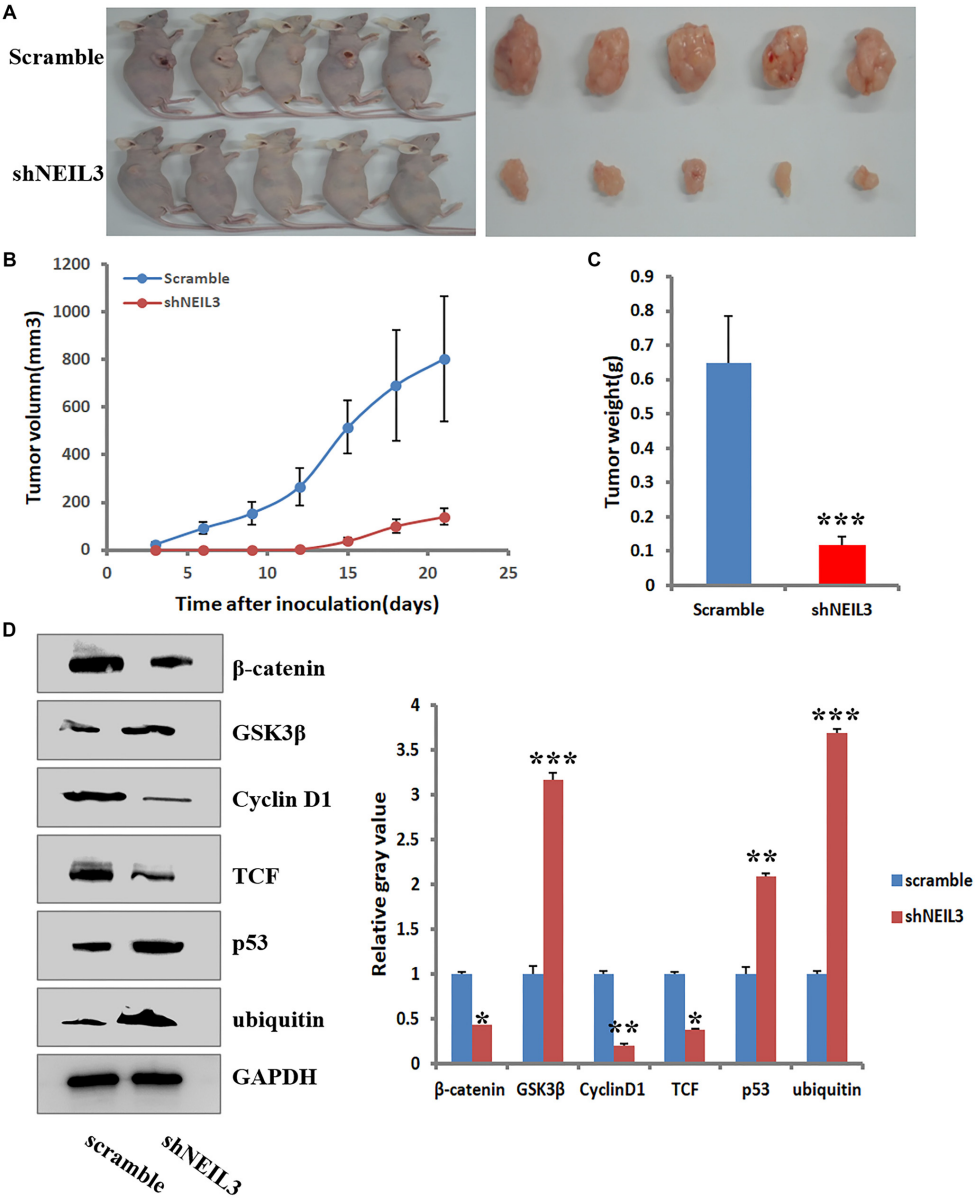
**Knockdown NEIL3 reduced tumor growth through WNT signaling pathway *in vivo*.** ECA109 cells were transfected with shNEIL3 or scramble and then injected into nude mice. At different days, mice were monitored, and implanted tumors were collected on day 21 of treatment (A). Effects of shNEIL3 on tumor volume at different time points (B) and tumor weight at day 25 (C). Tumor at day 25 was collected and Western Blot was determined on effects of NEIL3 on WNT signaling pathway (D) and their data analysis (E). *P < 0.05, **P < 0.01, ***P < 0.001. Significant difference, compared to scramble. NEIL3: Nei like DNA glycosylase 3.

The impact of NEIL3 on EC was investigated through *in vivo* experiments using shRNA-mediated knockdown techniques. NEIL3 knockdown resulted in a significant reduction in both tumor volume (Figure 5A) and weight (Figure 5B). Additionally, suppression of NEIL3 led to a marked decrease in the protein expression of β-catenin, cyclin D1, and TCF within the tumors (Figure 5C and 5D), while expression levels of GSK3β, p53, and ubiquitin proteins increased notably (Figure 5C and 5D). These findings underscore the critical role of NEIL3 in EC progression and highlight its potential as a therapeutic target for intervention.

## Discussion

EC is characterized by genomic instability and aberrant DNA repair processes, highlighting the importance of understanding NEIL3’s role in disease progression. Our findings show that NEIL3 downregulation significantly reduces tumor volume and weight, suggesting a pro-tumorigenic function in EC. TOP2A, an essential enzyme for DNA replication and transcription, is also implicated in cancer progression, including EC [19]. Targeting TOP2A has demonstrated therapeutic potential in various malignancies [20]. However, despite the well-established individual roles of TOP2A and NEIL3, their relationship remains unexplored. TOP2A encodes topoisomerase IIα, which modulates DNA topology to facilitate replication, transcription, and repair. Its overexpression is associated with multiple tumors [11, 12, 14], underscoring its significance in tumorigenesis. NEIL3, in contrast, plays a key role in the base excision repair pathway, maintaining genomic stability and preventing mutations [21, 22]. Our results indicate that both NEIL3 and TOP2A are upregulated in the EC cell lines examined, suggesting their involvement in disease progression. This upregulation may contribute to increased cancer cell proliferation and survival, aligning with previous studies linking these factors to tumorigenesis. Investigating the interaction between NEIL3 and TOP2A could provide valuable insights into the molecular mechanisms driving EC, potentially identifying novel therapeutic targets or diagnostic markers. Further experimental studies are needed to validate any association between these genes, which could improve disease management.

We used ECA109 EC cells to evaluate the effects of NEIL3 overexpression and TOP2A knockdown on proliferation, colony formation, migration, invasion, and apoptosis. Our results showed that NEIL3 overexpression significantly enhanced proliferation, colony formation, migration, and invasion while reducing apoptosis. In contrast, TOP2A knockdown inhibited these functions and promoted apoptosis. Furthermore, we found that NEIL3 overexpression could not reverse the effects of TOP2A knockdown. In our previous analysis using the STRING database, we identified a potential association between NEIL3 and TOP2A, prompting us to further explore their relationship. Surprisingly, when we investigated whether NEIL3 overexpression or knockdown affected TOP2A expression levels, we found that TOP2A expression remained unchanged. Likewise, TOP2A expression had no effect on NEIL3 levels. This suggests that TOP2A may inhibit clonogenesis through a NEIL3-independent mechanism. Although both NEIL3 and TOP2A play critical roles in DNA metabolism, our findings suggest that their functional mechanisms and pathways of action may operate independently. Additionally, we found that NEIL3 overexpression increased β-catenin, TCF, and cyclin D1, while decreasing GSK3β, p53, and ubiquitin levels. In contrast, TOP2A knockdown reduced β-catenin, TCF, cyclin D1, p53, and ubiquitin but did not affect GSK3β levels. Notably, NEIL3 overexpression did not counteract the decreased β-catenin, TCF, and cyclin D1 levels caused by TOP2A knockdown, although it did modulate ubiquitin and p53 expression. Specifically, NEIL3 overexpression reversed the increase in p53 expression induced by TOP2A knockdown.Given p53’s role as a tumor suppressor involved in cell cycle arrest, apoptosis, and DNA repair, our findings suggest that NEIL3 could modulate p53 expression, potentially influencing cell survival decisions in EC cells. This highlights a complex regulatory relationship between NEIL3, TOP2A, and WNT signaling pathways in ECA109 EC cells.

The interactions between NEIL3, TOP2A, and WNT signaling align with established knowledge, but our study provides new insights in the context of EC. The WNT pathway is well known for regulating cellular processes, such as proliferation and differentiation, with aberrant activation contributing to cancer development through β-catenin stabilization and cyclin D1 upregulation [23]. Our findings support this, demonstrating that NEIL3 overexpression increases β-catenin and cyclin D1 levels, potentially promoting cell proliferation and tumor growth. Additionally, our data suggest a potential link between NEIL3 and the ubiquitin-proteasome system (UPS), a key regulator of protein degradation and turnover. NEIL3 overexpression led to reduced ubiquitin levels, implying a role in protein stability regulation. However, the persistence of elevated ubiquitin levels following TOP2A knockdown, despite NEIL3 overexpression, suggests a more complex mechanism that warrants further investigation.

## Conclusion

In summary, our study uncovers novel regulatory mechanisms involving NEIL3, TOP2A, and WNT signaling in EC. Our findings suggest that NEIL3 may influence tumor growth through pathways associated with β-catenin, p53, and the UPS. Further investigation into these mechanisms could reveal new therapeutic opportunities for EC and other malignancies characterized by dysregulated DNA repair and WNT signaling. However, a key limitation of our study must be acknowledged: we assessed the effects of NEIL3 and TOP2A in only one EC cell line. While our results provide valuable insights, this reliance on a single model restricts the generalizability of our findings and may limit their broader relevance.

## Data Availability

The data used to support the findings of this study are available from the corresponding author upon request.
